# Effect of Serelys Homme on the Incidence and Severity of Vasomotor Symptoms and Quality-of-Life Impairments in Patients Receiving Hormone Therapy and Radiation for Localized Prostate Cancer: Results of the ESCULAPE Phase 2 Prospective Study

**DOI:** 10.1016/j.adro.2023.101255

**Published:** 2023-04-24

**Authors:** Yazid Belkacemi, Gabriele Coraggio, Anne Brunel, Annie Jouhaud, Alexandre Ingels, Charlotte Joly, Asma Hadhri, Wissal Hassani, Gokoulakrichenane Loganadane, Carolina Saldana, Nabila Ouidir, Barbara Vega, Kamel Debbi, Alexandre de La Taille

**Affiliations:** a Assistance Publique - Hôpitaux de Paris (AP-HP), Radiation Oncology Department and Henri Mondor Breast Center, Henri Mondor University Hospital, Créteil, France; b Institut national de la santé et de la recherche médicale (INSERM), Unit 955 (i-Bio), Institut Mondor de Recherche Biomédicale, University of Paris-Est Créteil (UPEC), Créteil, France; c Biostatistics, Stateo, Fontvieille, Monaco; d Urology Department, Henri Mondor University Hospital, University of Paris-Est Créteil (UPEC), Créteil, France; e Medical Oncology Department, Henri Mondor University Hospital, Créteil, France; f Pathology Department, Henri Mondor University Hospital, Créteil, France; g Medical Department, Sérélys Pharma, Fontvieille, Monaco

## Abstract

**Purpose:**

Androgen deprivation therapy (ADT) may cause vasomotor symptoms (VMS) including hot flushes and sweats, which affect quality of life (QoL). Serelys Homme is a nonhormonal and a natural origin product that could affect VMS in men undergoing ADT. We evaluated effectiveness and tolerance of Serelys Homme administration on VMS and QoL of patients undergoing combined ADT and radiation therapy for prostate cancer.

**Methods and Materials:**

Between April 2017 and July 2019, 103 patients were screened, and 53 patients refused to participate in the study. Serelys Homme therapy consisted of a daily administration of 2 tablets for 6 months. Patients were evaluated with 4 questionnaires including the adapted Modified Rankin Scale (adapted-MRS), European Quality of Life 5 Dimensions 3 Level Version (EQ 5D3L), Functional Assessment of Cancer Therapy–Prostate (FACT-P), and Hot Flash Related Daily Interference Scale (HFRDIS) at day 0, day 90 (D90), and day 180 (D180). Statistical evaluation was performed using the Wilcoxon rank sign test. A 2-sided *P* < .05 was considered statistically significant.

**Results:**

Among the 50 patients, 4 withdrew after inclusion. All patients (n = 46) received either postoperative or definitive radiation therapy combined with a short (n = 15) or long course (n = 31) of ADT. Serelys Homme administration significantly decreased the rate of patients who had ≥7 VMS and 3 to 6 VMS per day. The number of patients presenting with moderate or severe VMS was decreased at D90 (*P* = .005) and at D180 (*P* = .005). In addition, VMS duration was reduced at D90 (*P* = .002) and D180 (*P* < .001). Finally, at D90 and D180, 11.1% and 16.0% of patients, respectively, with initial severe or moderate VMS had a complete response without further symptoms. Among QoL parameters, fatigue decreased significantly. Effectiveness evaluated by doctors was rated as moderate or good to excellent VMS control in 20% and 60% of the patients, respectively. No side effects were recorded in the whole population.

**Conclusions:**

This study demonstrated effectiveness and excellent tolerance of Serelys Homme. We observed a significant reduction of the frequency, duration, and intensity of hot flushes and sweats induced by ADT. Serelys Homme increased QoL scores. These encouraging results open the prospect to further studies and Serelys Homme use in patients undergoing ADT for prostate cancer.

## Introduction

Androgen deprivation therapy (ADT) has been increasingly used to enhance patients’ outcomes either after short- or long-term hormone therapy (HT) combined with radiation therapy (RT) for organ-confined disease or as standard treatment for men with metastatic prostate cancer (PCa). HT in PCa is associated with several side effects including vasomotor symptoms (VMS) (in particular, hot flushes), sexual dysfunction and gynecomastia, osteoporosis and other metabolic disorders, depression syndrome and neurocognitive deficits, thromboembolic disease, and cardiovascular disease. All these side effects can highly affect quality of life (QoL) and observance of long-term therapy duration.^1^

Hot flushes are considered one of the most common and bothersome side effects during HT. For example, in patients under gonadotropin-releasing hormone agonists, 80% of patients will develop hot flushes, and about one-fourth of them consider this symptom as the most significant side effect.^2^ In daily practice, a large number of patients who cannot acclimate their bodies to the low testosterone state will need specific therapies including gabapentin and venlafaxine or low-dose estrogen and progestin.^3^^,^^4^ However, the benefits of this option need to be weighed against the thromboembolic risk associated with estrogen use.^5^ Thus, although some of these drugs have been reported to be effective for hot flushes,^3^^,^^6^ the best treatment for hot flushes for men treated with HT is unclear.

Serelys Homme is a nonhormonal and plant-based product composed of purified and specific cytoplasmic pollen extracts that could affect VMS control in men undergoing HT. Purified and specific cytoplasmic pollen extract, known by the brand name of PureCyTonin, has been evaluated in several preclinical and clinical studies, where it demonstrated its value as a safe and nonestrogenic alternative for alleviating hot flushes in menopause. These reports concluded that pollen extract is a nonestrogenic alternative for managing menopausal symptoms in cancer survivors.^7^, ^8^, ^9^

We aimed to evaluate effectiveness and tolerance of Serelys Homme administration on VMS and QoL of patients undergoing combined ADT and RT for PCa.

## Methods and Materials

### Study design

This study was approved by our ethical committee and conducted in accordance with the Declaration of Helsinki (as revised in 2013). All patients enrolled completed the informed consent form. The product Serelys Homme used for this study was provided by Serelys Pharma, Monaco. Serelys Homme is composed of 320 mg pollen/pistil extract, with a recommended daily dose of 320 mg. The product is available under different commercial names besides Serelys Homme. This prospective single-arm study evaluated the efficacy of Serelys Homme on hot flushes among patients with PCa receiving combined HT and RT. The primary endpoint was the efficacy in reducing the strength, frequency, and duration of hot flushes. The secondary endpoint was its efficacy in improving QoL.

Four questionnaires were used: (1) the adapted Modified Rankin Scale (adapted-MRS; women menopausal rating scale) specifically evaluates the characteristics of hot flushes; (2) the European Quality of Life 5 Dimensions 3 Level Version (EQ 5D3L) scale focuses on global QoL; (3) the Functional Assessment of Cancer Therapy – Prostate (FACT-P) investigates physical, social, emotional, functional, and psychological well-being; and (4) the Hot Flash Related Daily Interference Scale (HFRDIS) investigates the effect of hot flushes on daily activities. Evaluation was made at day 0 (D0), day 90 (D90), and day 180 (D180).

### Study population

Factors determining eligibility in patients with PCa receiving ADT at Henri Mondor University Hospital who had hot flushes included desire for treatment, performance status of 0 to 2, a prognosis of 1 year or more, and the ability to take the drug orally. We excluded patients with severe mental illness, serious known liver or renal dysfunction, or any current use of other herbal medicine, selective serotonin reuptake inhibitors, or serotonin-noradrenalin reuptake inhibitors.

**Figure 1:**
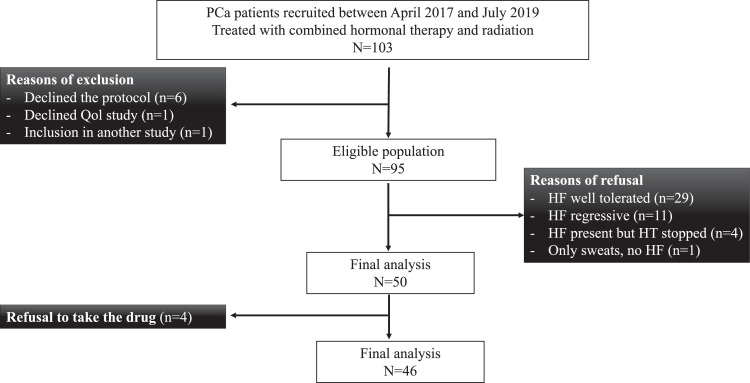
Flowchart of eligible patients selected for this study. *Abbreviations:* HF = hot flush; HT = hormone therapy; PCa = prostate cancer; QoL = quality of life.

Between April 2017 and July 2019, 103 patients were screened, and 53 patients refused to participate to the study, mainly (78%) considering their symptoms as limited or regressive. In Fig. 1, we present the flowchart of the study.

### Study protocol

All patients who underwent RT HT for PCa were screened at a baseline visit. Patient characteristics, including age, body weight, body mass index, PCa status (clinical stage, Gleason score, and duration of cancer), details of ADT (gonadotropin-releasing hormone agonist/antagonist), and current complications, were recorded. After providing written informed consent, eligible patients received Serelys Homme therapy consisting of a daily administration of 2 tablets (each containing 160 mg pollen extract + 5 mg vitamin E) for 6 months. During the trial, all patients were asked to keep a daily report describing their hot flushes and compliance. The diary included information regarding the frequency of hot flushes, the number of underwear changes, and the number of tablets taken. The diary was collected by the attending doctors at each visit. In addition, patients were evaluated with the 4 questionnaires: adapted-MRS (women menopausal rating scale) with the maximum duration of the episode (<1 minute, ≥1 minute, and <5, or ≥5 minute), EQ 5D3L scale with a visual analog scale, FACT-P, and HFRDIS at D0, D90, and D180. Tolerance was assessed by the investigator at D90 and D180, and side effects were planned to be recorded using the National Cancer Institute Common Terminology Criteria for Adverse Events version 4.0 classification.

### Statistical analyses

Continuous variables were reported as mean ± SD, median, and interquartile range. Categorical variables were reported as counts and percentages. Statistical evaluation was performed using the Wilcoxon rank sign test on the change from inclusion. A 2-sided *P* < .05 was considered statistically significant.

All analyses were performed using SAS statistical software, version 9.4 (SAS Institute, Inc, Cary, NC)

## Results

### Participant and treatment characteristics

Among the 50 patients, 4 refused to take Serelys Homme after their inclusion. Thus, analyses concerned 46 patients. PCa risk was either high (n = 25) or intermediate (n = 21). Histology Gleason (G) score distribution was G6 (n = 4), G7 (n = 22), G8 (n = 8), G9 (n = 10), and G10 (n = 2). Patients received either postoperative or salvage RT (n = 31) or definitive RT (n = 15) combined with short (n = 15) or long course (n = 31) of HT. Sixteen and 30 patients had prostatic bed only (median, 66 Gy; range, 66-72 Gy) and pelvic irradiation (median, 70 Gy; range, 66-74 Gy), respectively (Table 1).

**Table 1:** Description of the population

Characteristics		Main population n = 46 (%)
Age (y)	Mean ± SD	67.3 ± 8.9
WHO BMI	Normal weight	15 (32.6)
	Overweight	24 (52.2)
	Obesity	7 (15.2)
Performance status	0	45 (97.8)
	1	1 (2.2)
Prostatectomy	Yes	23 (50)
	No	23 (50)
Delay from prostatectomy (y)	Median (Q1, Q3)	3.62 (1.5, 6.1)
Duration of hormone therapy	Short	15 (32.6)
	Long	31 (67.4)
Risk	Intermediate	20 (44.4)
	High	26 (65.6)
Gleason score	G6	4 (8.9)
	G7	22 (46.7)
	G8-10	20 (17.8)
Irradiation volumes	Prostatic bed	16 (35)
	Prostatic bed + pelvis	30 (63)
Dose to prostatic bed (Gy)	Median (Q1, Q3)	66 (66, 72)
Dose to pelvis (Gy)	Median (Q1, Q3)	70 (66, 74)

*Abbreviations:* BMI = body mass index; Q1 = first quartile; Q3 = third quartile; normal weight = [18.5,25]; overweight = [25,30]; obesity = ≥30 kg/m^2^; SD = standard deviation; WHO = World Health Organization.

### Efficacy results

**Figure 2:**
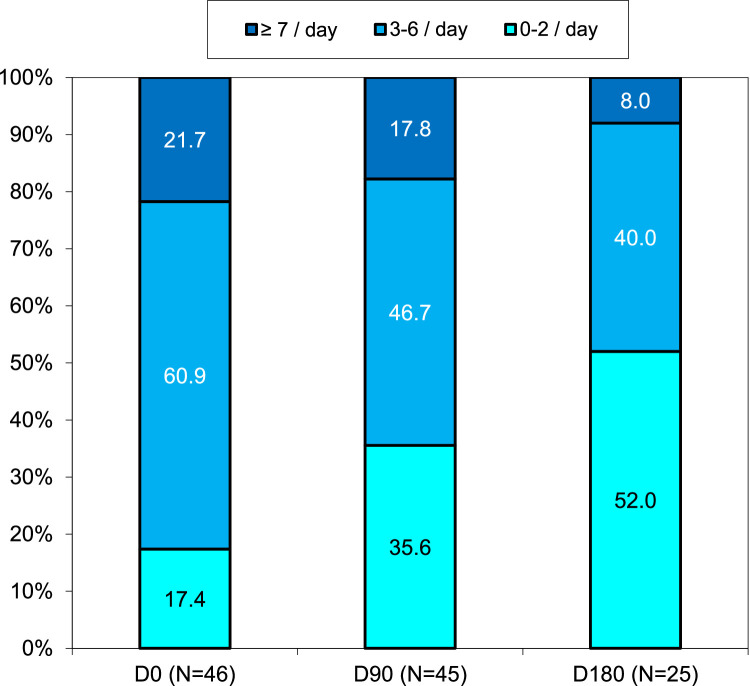
Frequency of vasomotor symptoms.

**Figure 3:**
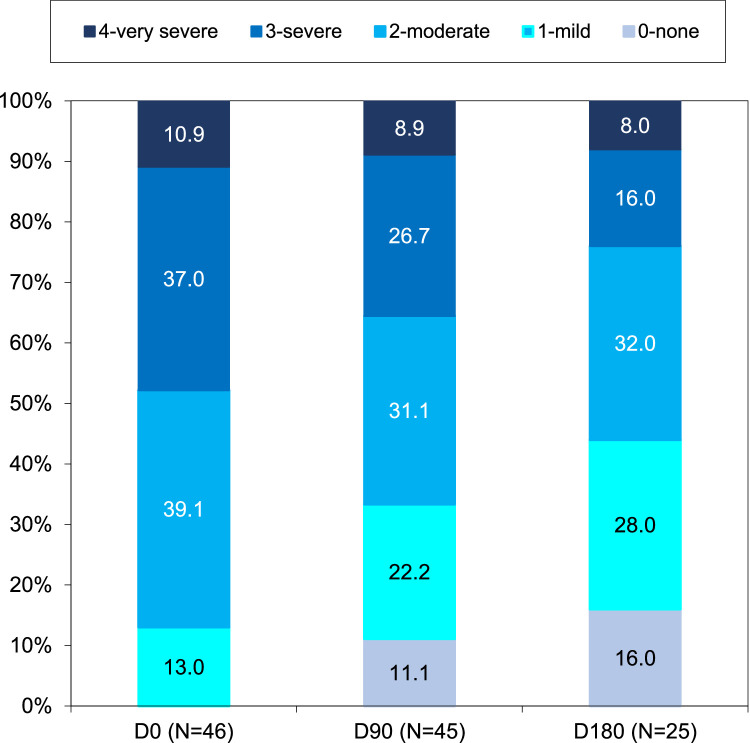
Intensity of vasomotor symptoms following menopause rating scale.

**Figure 4:**
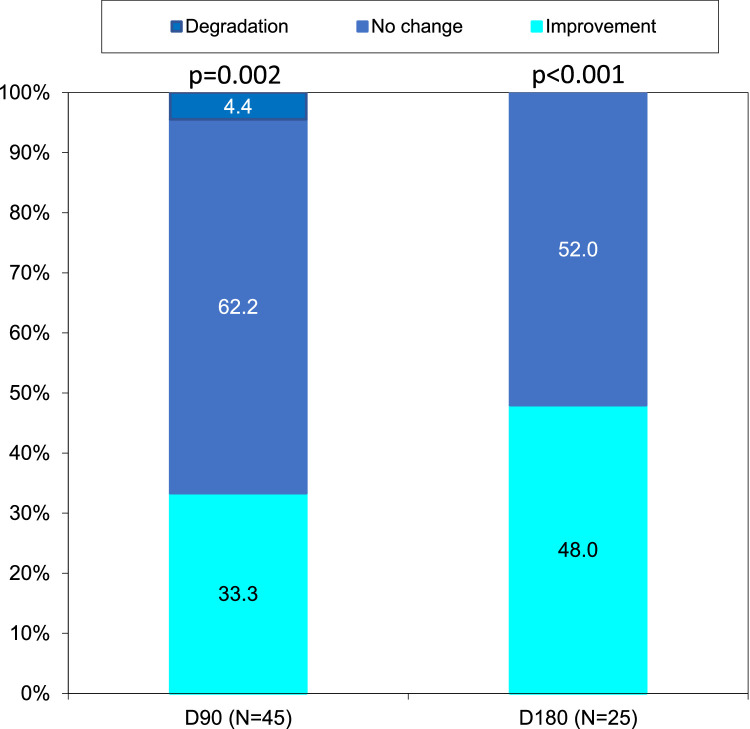
Vasomotor symptom duration.

Serelys Homme administration significantly decreased the rate of patients who had ≥7 VMS per day during follow-up (21.7% at D0, 17.8% at D90, and only 8.0% at D180). The rate of patients presenting 3 to 6 VMS also decreased 60.9%, 46.7%, and 40.0% at D0, D90, and D180, respectively (*P* = .055 at D90 and *P* = .02 at D180) (Fig. 2). Serelys Homme administration decreased the number of patients presenting moderate or severe VMS intensity at D90 (*P* = .005) and at D180 (*P* = .005) (Fig. 3) and reduced VMS duration at D90 (*P* = .002) and D180 (*P* < .001) (Fig. 4). Finally, at D90 and D180, 11.1% and 16.0% of patients, respectively, with initial severe or moderate VMS had a complete response without further symptoms with Serelys Homme (Fig. 3).

### QoL results

**Figure 5:**
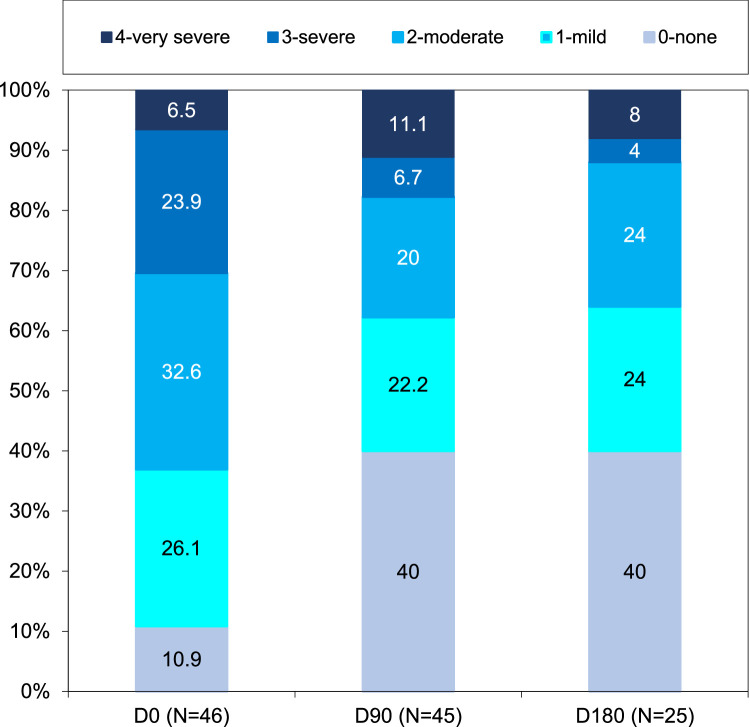
Fatigue linked to vasomotor symptoms.

**Figure 6:**
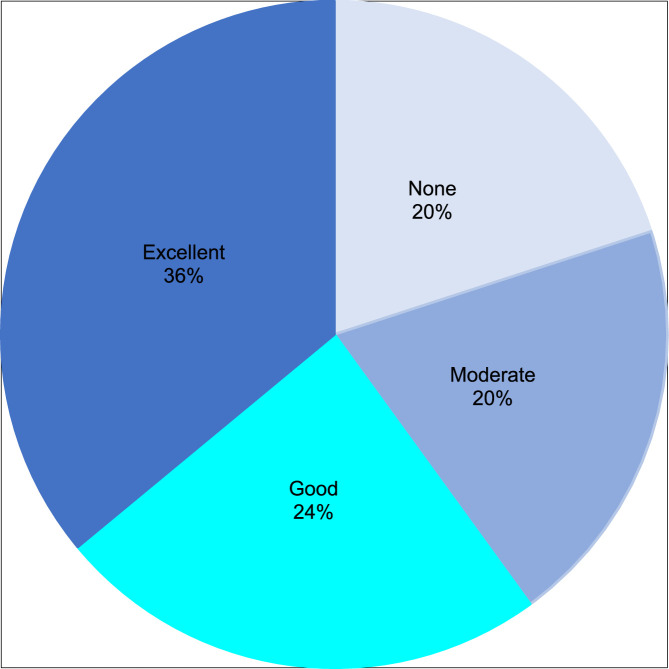
Efficacy on vasomotor symptoms control according to doctors’ evaluation.

In terms of QoL parameters, Serelys Homme administration significantly decreased fatigue at D90 (*P* = .006) and D180 (*P* = .002) (Fig. 5). After individual reports, all symptoms decreased during follow-up. Effectiveness evaluated by doctors was rated as moderate or good-to-excellent VMS control in 20% and 60% of the patients, respectively (Fig. 6).

### Safety results

No side effects were recorded in the whole population, and tolerance was considered as excellent by the investigator (n = 46 at D90 and n = 25 at D180).

## Discussion

The mechanisms that cause the disorder of hypothalamic thermoregulation resulting in hot flushes are not completely known.^10^, ^11^, ^12^, ^13^, ^14^ The reduction in testosterone is believed to affect the function of serotonin, noradrenalin, and beta-endorphins, leading to instability of the hypothalamus thermoregulatory center with consequent intermittent downregulating of body temperature through vasodilation and increased perspiration.^12^ Calcitonin gene–related peptide is a vasodilating neuropeptide excreted in augmented quantities in postmenopausal women with hot flushes but not in castrated men,^13^^,^^14^ suggesting some differences in hot-flush mechanisms between the 2 sexes.

Hot flushes are defined as an intense feeling of warmth, accompanied sometimes by facial flushing, sweating, chills, heart palpitations, nausea, and night sweats. Depending on their intensity and frequency, these symptoms may easily give rise to fatigue, feelings of anxiety, and sleep disturbances with an effect on adherence to HT.^10^ Many patients will not acclimate their bodies to the low testosterone state in the next months after HT initiation.

Hot flushes are frequently reported (34%-80%) in men undergoing HT for PCa, and many of them who experience hot flushes in the beginning of their treatment still report hot flushes at 5 years and 8 years (70% and 40%, respectively), often with the same frequency and intensity as the first months after HT.^15^ However, it is important to discuss the progression of VMS from D0 onward, reported in the randomized trials, that demonstrated minimal change in hot flushes for patients undergoing ADT who received placebo treatment. In Loprinzi et al's^4^ double-blind placebo-controlled clinical trial comparing gabapentin to placebo, hot-flush scores decreased in the placebo group by 4.1 units versus 3.2, 4.6, and 7.0 units in the 3 increasing dose (300, 600, or 900 mg/d) of gabapentin groups between the fourth treatment week and the baseline week. In addition, comparing the 3 gabapentin arms to the placebo arm did not result in significant hot-flash differences. In another phase 3 trial^3^ comparing 3 drugs (venlafaxine, cyproterone, medroxyprogesterone acetate), the change in median daily hot-flush scores between randomization and 1 month was significantly different among the drugs and particularly low with venlafaxine. Also, the decrease of daily hot flushes from baseline was significant for all 3 groups.

The relationship between hot flushes and anxiety is an important parameter to consider regarding therapy observance during follow-up of patients receiving ADT. In patients who do not experience persistent hot flushes, the distress decreases after 3 months; conversely, the presence of hot flushes keeps distress levels high, and many patients even choose to discontinue HT to reduce VMS.^12^^,^^16^

Our cohort of patients treated with Serelys Homme presented with an incidence of hot flushes comparable to literature (60.9% and 21.7% had 3-6 and ≥7 VMS per day, respectively, at D0). The reduction of strength, frequency, and intensity of VMS was significant at 3 months and even more accentuated at 6 months, with only 8.0% of patients presenting with ≥7 VMS per day at D180. Furthermore, 11.1% and 16.0% of patients with initial severe or moderate VMS, respectively, had a complete response without further symptoms at 6 months.

Among the published prospective trials presented in Table 2,^3^^,^^4^^,^^17^^,^^18^ it is not easy to compare results of one study to another given the importance of the methodological differences, in particular, in terms of study objectives/designs and the tools used to evaluate incidence, intensity, and duration of the hot flushes over different observation times. For example, in our study and in the 2 recent trials,^17^^,^^18^ the results at 1, 3, and 6 months were compared with the baseline, whereas in the earlier studies,^3^^,^^4^ follow-up was limited to only 4 weeks. At 3 and 6 months, the conclusions from our data were similar, with a decrease of hot-flush frequency and intensity, as among both randomized studies that used estradiol^17^ and fetal estrogen estetrol.^18^ The earlier trials^3^^,^^4^ conclude also in a reduction in hot flushes in the arms that tested gabapentin^4^ and the 3 investigated molecules (venlafaxine, cyproterone acetate, and medroxyprogesterone acetate) versus the placebo.^3^ Interestingly, only our study included patients who received either combined postoperative or salvage RT (n = 31) or definitive RT (n = 15) combined with a short (n = 15) or long course (n = 31) of ADT, while the others included more advanced PCa.

**Table 2:** Summary of prospective randomized trials designed to evaluate treatment efficacy of different agents on vasomotor HF in men undergoing ADT for prostate cancer

Study	Study design	Number of patients	Drugs and compounds	Evaluation	Results	Comments and conclusion
Loprinzi et al^4^ (2009)	Phase 3, double-blind, placebo-controlled trial	214	Gabapentin 300 (n = 54) vs 600 (n = 53) vs 900 (n = 54) mg/d Placebo (n = 53)	Daily record of HF at baseline week vs 4 treatment weeks Decrease of MHF scores	3.2 units 4.6 units 7 units vs 4 units (placebo)	Highest dose of gabapentin decreases HF, to a moderate degree, in men with ADT-related HF
Irani et al^3^ (2010)	Phase 3, double-blind, controlled trial (n = 311)	311	V (n = 102) CA (n = 101) MA (n = 108)	MHF score: change at 1 mo after randomization	47.2% (IQR, –74.3 to –2.5) 94.5% (IQR, –100 to –74.5) 83.7% (IQR, –98.9 to –64.3)	Decrease from baseline larger in CA and MA vs V As CA interferes with ADT, MA could be the standard
Russel et al^17^ (2022)	Phase 3, double-blind, placebo-controlled trial	78	Estradiol (0.1%) 0.9 mg/d (n = 39) Placebo (n = 39)	Weekly record of HF at baseline and 1, 3, and 6+ mo MAD questionnaire of QoL	Daily MAD of HF score = –1.6 (*P* = .04); per protocol: –2.2 (*P* = .001) Weekly MAD of HF score = –19.6 (*P* = .11); per protocol: –27 (*P* = .02) No effect of QoL	Estradiol reduces HF frequency in men undergoing ADT Transdermal estradiol could be considered for men with burdensome HF when other treatments have failed Risk of breast effects and fat gain should be considered
Zimmerman et al^18^ (2022)	Phase 2, double-blind, placebo-controlled trial	62	E4 (n = 41) Placebo (n = 21)	Effect on HF Q-Man, FACT-P questionnaire for HRQL	Effect of E4 on HF prevention at 24 wk (14.3% vs 60%, *P* < .001) Lower incidence of night sweats, arthralgia, and fatigue but more gynecomastia Improve HRQL scores with E4	For patients on ADT, cotreatment with a high dose of E4 almost completely prevents HF and improves QoL but increases nipple sensitivity and gynecomastia
ESCULAPE study (current report)	Phase 2 single-arm study	46	SH	Effect of SH on HF at 3 and 6 mo 4 questionnaires for QoL including adapted-MRS, EQ 5D3L, FACT-P, and HFRDIS	SH decreased the rate of patients with ≥7 HF and 3-6 HF per day Duration of HF reduced by SH Among QoL parameters, fatigue decreased after SH	Effectiveness of SH is demonstrated with a significant reduction of the HF frequency, intensity, and duration with a positive effect on QoL

*Abbreviations:* adapted-MRS = adapted Modified Rankin Scale; ADT = androgen deprivation therapy; CA = cyproterone acetate; E4 = fetal estrogen estetrol; EQ 5D3L = European Quality of Life 5 Dimensions 3 Level Version; FACT-P = Functional Assessment of Cancer Therapy–Prostate; HF = hot flushes; HFRDIS = Hot Flash Related Daily Interference Scale; HRQL = health-related quality of life; IQR = interquartile range; MA = medroxyprogesterone acetate; MAD = mean adjusted difference; MHF = median hot-flush score; Q-Man = questionnaire for evaluation of the effect on estrogen- and androgen-deficiency symptoms; QoL = quality of life; SH = Serelys Homme; V = venlafaxine.

Besides VMS including hot flushes, several studies evaluated the effect of these symptoms on QoL using different scales. In our study, an exhaustive evaluation was based on 4 questionnaires. Like for the other agents,^17^^,^^18^ we observed a greater positive effect of Serelys Homme on fatigue (Fig. 5). QoL parameters like fatigue, as well as the intensity of all symptoms, decreased accordingly in our study. Conversely, the 2 earlier studies were unable to assess QoL or parameters other than the frequency of hot flushes at 1 month.

Hot flushes and other VMS are also important side effects of HT in breast cancer. Therapeutic management of VMS is largely extrapolated from patients with breast cancer receiving tamoxifen. Randomized clinical trials have demonstrated efficacy of gabapentin and venlafaxine; these agents may be used for this off-label indication in men receiving HT.^3^^,^^4^^,^^19^ Low-dose estrogen and progestins can also be considered,^19^^,^^20^, ^21^ but the benefits of this option need to be weighed against the thromboembolic risk associated with estrogen use.^22^ Additionally, acupuncture has been shown to be of potential benefit.^23^ Conservative strategies to abort hot flushes include sleeping with an open window or drinking cold beverages. Lastly, regular exercise can help decrease frequency and severity of hot flushes; given the benefit of exercise for other ADT side effects and overall health, this recommendation is strongly encouraged. Overall, a stepwise approach is suggested for hot-flush management, including exercise, conservative measures, use of venlafaxine or gabapentin, and lastly use of estrogen or progestins as needed while weighing the potential risks versus benefits of each treatment option.

The mechanisms for controlling hot flushes using all these agents and techniques are very different with more or less effectiveness. Purified and specific cytoplasm pollen extract, PureCyTonin (or Serelys), a nonhormonal option to menopausal HT, has proven efficacious in alleviating hot flushes and other symptoms such as sleep disorders and irritability. From the mechanisms point of view, serotonin, norepinephrine, and other neurotransmitters are implicated in the generation of hot flushes. Thus, selective serotonin reuptake inhibitors and selective serotonin-norepinephrine reuptake inhibitors such as paroxetine, fluoxetine, and venlafaxine are effective for the treatment of hot flushes by acting on serotonergic neurons. Apple et al^24^ suggested serotonin reuptake as a part of mode of action of Serelys in a rat brain model. This could have an amplifying effect in other brain neurotransmitter pathways that control thermoregulation (for hot flushes), sleep, and mood, which depend of the decrease or the lack^25^ of estrogen during the female menopausal transition.^26^ In the context of PCa, the administration of ADT leads to androgen deprivation that we can easily compare with the mechanisms described for the menopausal transition of women or during HT for breast cancer.

Beside efficacy, Serelys safety is demonstrated for women with VMS during menopause and HT for breast cancer.^8^ In our study of patients with PCa, tolerance as evaluated by the investigators was excellent in all our patients at D90 and D180. Our excellent safety results with Serelys Homme are in accordance with the clinical data from the studies with Serelys for women that has the same formulation. In the review published by Genazzani et al,^9^ safety of Serelys in several studies has been clearly confirmed. Moreover, the finished product Serelys with a daily dose of 320 mg PureCyTonin and 10 mg vitamin E, which is the same daily dose as Serelys Homme, has been evaluated in a randomized double-blind placebo-controlled trial in women that did not show any significant changes in hormones levels supporting the nonhormonal action of the product.^9^

They are some limitations in our study regarding the design of the trial without a comparator arm and the small sample size. Our study is an open, phase 2, single-arm trial, not a controlled study with a placebo arm. The aim was only to evaluate safety and efficacy of Serelys Homme in patients with PCa undergoing ADT. Without any information on the efficacy in men, no sample size could be calculated.

## Conclusion

This study demonstrated effectiveness and excellent tolerance of Serelys Homme. We observed a significant reduction of the frequency, duration, and intensity of hot flushes and sweats induced by HT. Serelys Homme increased QoL scores. The use of nonpharmacologic and nonhormonal treatment as Serelys Homme could be an option for treating hot flushes in men. These encouraging results open the prospect to further studies and Serelys Homme use in patients undergoing HT for PCa.

## Disclosures

The authors declare that they have no known competing financial interests or personal relationships that could have appeared to influence the work reported in this paper.
